# GATA binding protein 5-mediated transcriptional activation of transmembrane protein 100 suppresses cell proliferation, migration and epithelial-to-mesenchymal transition in prostate cancer DU145 cells

**DOI:** 10.1080/21655979.2021.2018979

**Published:** 2022-03-31

**Authors:** Jiaolin Liu, Fanlu Lin, Xin Wang, Chaopeng Li, Qiangyuan Qi

**Affiliations:** a Department of Urology, The Central Hospital of Linyi, Linyi, Shandong, China; b Department of Urology, Linyi People’s Hospital, Linyi, Shandong, China

**Keywords:** Prostate cancer, transmembrane protein 100, GATA binding protein 5, transcription, migration, epithelial-to-mesenchymal transition

## Abstract

It has been reported that transmembrane protein 100 (TMEM100) acts as a tumor regulator in several types of cancers. However, whether the expression of TMEM100 is associated with the development and prognosis of prostate cancer (PCa) remains elusive. Therefore, the present study aimed to uncover the role of GATA binding protein 5 (GATA5)-mediated activation of TMEM100 in the proliferation, migration and epithelial-to-mesenchymal transition (EMT) of PCa cells. The expressions of TMEM100 and GATA5 in PCa patients were analyzed by the GEPIA database. The binding site of GATA5 and TMEM100 promoter was predicted by the JASPAR database. Expressions of TMEM100 and GATA5 in PCa cells were detected by qRT-PCR and Western blot analysis. Cell Counting Kit 8 and colony formation assays were performed to measure cell proliferation. In addition, cell migration, invasion and the expression of EMT-associated proteins were evaluated using wound healing, transwell assay and Western blotting assays, respectively. The bioinformatics analysis revealed that TMEM100 was downregulated in PCa and was associated with overall survival of PCa. In addition, TMEM10 overexpression attenuated cell proliferation, migration, invasion and EMT in PCa cells. The interaction between TMEM100 and GATA5 was verified using dual luciferase reporter and chromatin immunoprecipitation assays. Furthermore, the results showed that GATA5 was downregulated and GATA5 silencing reversed the inhibitory effects of TMEM10 on PCa cells. Overall, the current study suggested that the GATA5-mediated transcriptional activation of TMEM100 could affect the behavior of PCa cells and was associated with poor prognosis in PCa.

## Introduction

Prostate cancer (PCa) is the fifth leading cause of cancer-related mortality and the second most common type of cancer among men worldwide [1,2]. The morbidity and mortality of PCa are closely associated with age and the highest incidence has been observed in elderly men, >65 years of age [3]. At present, advanced therapeutic technology, including androgen ablation and surgical treatment, has been applied to treat PCa [4,5]. However, the side effects and sequelae of surgery and drugs may lead to a decline in patients’ quality of life [6,7]. Therefore, it is urgently needed to explore the molecular mechanism underlying the initiation and progression of PCa for the development of novel diagnostic and therapeutic approaches.

Transmembrane protein 100 (TMEM100), a gene located within the 17q32 locus, encodes a 134-amino acid protein with two predicted transmembrane domains (amino acids 53–75 and 85–107) [8]. TMEM100 is highly conserved among vertebrates, including mammals, zebrafish and chickens, and it is structurally unique from any known family of proteins [9]. It has been reported that TMEM100 is associated with cancer tumorigenesis and progression [10]. Han et al [11] showed that the expression of TMEM100 was significantly decreased in non-small cell lung cancer (NSCLC) tissues, while TMEM100 overexpression in NSCLC cell lines could inhibit cell proliferation, invasion and migration. In addition, Zhuang et al [12] revealed that TMEM100 overexpression could reduce the migration and invasion abilities of gastric cancer cells and enhance their sensitivity to chemotherapy. Data from GEO database also show that TMEM100 expression is down-regulated in PCa (GSE69223). However, the biological role of TMEM100 in PCa has not yet been fully investigated.

The GATA protein family (GATA1-6) is comprised of transcription factors that bind to the DNA consensus GATA sequence [13]. GATA transcription factors have been documented to suppress the malignant transformation of various types of human cancer [14,15]. For example, depletion of GATA binding protein 5 (GATA5) expression causes the proliferation and colony formation in hepatocellular carcinoma cells, but upregulated GATA5 suppresses the growth and metastasis of cholangiocarcinoma cells [16,17].

The current study aimed to investigate the effects of TMEM100 expression on PCa progression and uncover the potential mechanism underlying the effect of TMEM100 on regulating the development of PCa. More specifically, the present study evaluated the effect of TMEM100 on the proliferation, migration and epithelial-to-mesenchymal transition (EMT) of PCa cells and the combination with GATA5 in PCa.

## Materials and methods

### Bioinformatic analysis

The expressions of TMEM100 and GATA5 in PCa were analyzed by the GEPIA database (http://gepia.cancer-pku.cn) [18]. The relationship between TMEM100 and overall survival in patients with PCa was also characterized by the GEPIA database. In addition, the binding site of GATA5 to TMEM100 promoter was predicted by JASPAR website (http://jaspar.genereg.net/) [19].

### Cell culture

The normal human prostatic epithelial cell line, P69, and the PCa cell lines, LNCap, PC3, DU145 and 22Rv1, were obtained from the American Type Culture Collection. The cells were maintained in DMEM (Gibco; Thermo Fisher Scientific, Inc.) supplemented with 10% FBS and 1% penicillin/streptomycin in a humidified incubator at 37°C and 5% CO_2_.

### Cell transfection

The pc-DNA3.1 vector containing the whole length of TMEM100 (Oe-TMEM100), GATA binding protein 5 (GATA5)-specific pcDNA overexpression plasmid (Oe-GATA5) and negative control empty pcDNA (Oe-NC), short hairpin (sh) RNA targeting GATA5 (sh-GATA5-1/2) and the corresponding negative control (sh-NC) were synthesized by Shanghai GeneChem Co., Ltd. The sequence for sh-GATA5-1: 5′- GCCTCTACCACAAGATGAA-3′. The sequence for sh-GATA5-2: 5′-GGGTTGGATGATACCTTAA-3′. The sequence for shRNA-NC: 5′- CCGGCAACAAGATGAAGAGCACCAACTC-3′. Cells were transfected with the recombinants using Lipofectamine® 2000 (Invitrogen; Thermo Fisher Scientific, Inc.) according to the manufacturer’s instructions.

### Reverse transcription-quantitative PCR (RT-qPCR)

Total RNA was extracted from transfected or untransfected cells using TRIzol® reagent (Invitrogen; Thermo Fisher Scientific, Inc.). RNA was reverse transcribed into first-strand cDNA using the PrimeScript RT Master Mix (Takara Bio, Inc.). cDNA was amplified using a SYBR PrimeScript RT-PCR kit (Takara Bio, Inc.) in an ABI PRISM 7900 Real-Time system (Applied Biosystems, Foster City, CA, USA). The PCR program was 95°C for 3 min, followed by 35 cycles of denaturation at 95°C for 30 s, annealing at 60°C for 30 s and extension at 72°C for 1 min. A final extension step at 72°C for 7 min was performed in each PCR assay. The primer sequences for PCR are presented as below: TMEM100, 5ʹ-TACCGAGCTCTCCTGCTACC-3ʹ (forward) and 5′-CCAGGCCAAAGATGGAGATA-3′ (reverse); GATA5, 5ʹ- AGTGCGAGCGGGACACGGTT-3′ (forward) and 5′-GAGCACTCACCAGCGGGCAG-3′(reverse); GAPDH: 5′-GGGAAACTGTGGCGTGAT-3′ (forward) and 5′-GAGTGGGTGTCGCTGTTGA-3′ (reverse). The relative expression levels of the target genes were normalized to GAPDH and calculated using the 2^−ΔΔCq^ method [20].

### Cell counting kit 8 (CCK-8) assay

The cell proliferation capacity of PCa cells transfected with Oe-TMEM100 or co-transfected with sh-GATA5 was measured using a CCK-8 assay [21]. Briefly, transfected DU145 cells were seeded into 96-well plates at a density of 5 × 10^3^ cells/well. Subsequently, following incubation for 24, 48 or 72 h, each well was supplemented with 10 μl CCK-8 reagent and cells were incubated for an additional 3 h. The absorbance at a wavelength of 450 nm was measured with a microplate reader (Anthos Zenyth 200 RT, Biochrom LTD, Cambridge, UK). All experiments were done in triplicate and each group was analyzed six times.

### Colony formation assay

Transfected cells were seeded into 6-well plates at a density of 500 cells/well and cultured for two weeks in DMEM supplemented with 10% FBS at 37°C. Subsequently, cells were fixed with 4% paraformaldehyde and stained with 0.1% crystal violet. Colonies were then imaged and counted under a light microscope (IX73; Olympus Corporation). Only colonies with >50 cells were counted. All experiments were repeated three times and each group was analyzed five times [22].

### Wound healing assay

Cells transfected with Oe-TMEM100 in the presence or absence of sh-GATA5 were seeded into 6-well plates and then incubated until they reached 90% confluency in DMEM with 10% FBS at 37°C. A pipette tip was used to create a single, straight scratch in the cell monolayer and the cells were then incubated for 24 h. The wound surface and the number of migratory cells were measured under an inverted microscope (BX51; Olympus Corporation) in five randomly selected fields for each well [23]. The migration rate was calculated based on the formula: (wound width at 0 h – wound width at 24 h)/wound width at 0 h × 100%.

### Cell invasion assay

Transfected cells were collected and suspended in serum-free DMEM at a final concentration of 2 × 10^5^ cells/ml. Subsequently, the cell suspension was loaded into the upper well of the Transwell chamber (Costar; Corning, Inc.) precoated with 0.1 ml Matrigel (BD Biosciences). DMEM with 10% FBS was added into the lower compartment. Following incubation for 24 h, cells on the upper chamber were wiped off with a cotton swab, while those on the lower one were fixed with 100% methanol, stained with hematoxylin and eosin and counted under a microscope (IX73; Olympus Corporation). Each assay was carried out at least three times [24].

### Luciferase reporter assay

Luciferase reporter assay was performed to verify the interaction between TMEM100 and GATA5 [25]. Briefly, The wild-type (WT) and corresponding mutational (MUT) fragments of TMEM100 promoter covering predicted DR1 sites were subcloned into a pGL3-basic vector (Promega Corporation) to construct the TMEM100-WT or TMEM100-MUT reporter plasmid, respectively. Subsequently, DU145 cells were co-transfected with luciferase reporter vectors and Oe-NC/Oe-GATA5 using Lipofectamine 2000. At 48 h post-transfection, the relative luciferase activity was measured using a Dual-Luciferase Reporter assay (Promega Corporation).

### Chromatin immunoprecipitation assay (ChIP)

ChIP assay was performed to evaluate the binding capacity of GATA5 to the TMEM100 promoter using RT-qPCR as previously described [26]. The cells were cross-linked with 1% formaldehyde for 10 min at 37°C and quenched with 2.5 M glycine for 5 min at room temperature. Following ultrasound rupture of chromatin, DNA was immunoprecipitated from cell lysates using anti-GATA5 antibody (dilution, 1:200; cat. no.sc-515,422; Santa Cruz Biotechnology, Inc.) and normal rabbit IgG (dilution, 1 µg/ml; cat. no. ab171870; Abcam). The precipitate of the crosslinked protein-DNA complexes was collected for DNA purification followed by verification employing qPCR. A nonspecific antibody against IgG served as a negative control.

### Western blot analysis

Total proteins were extracted from transfected cells with RIPA buffer (Auragene Bioscience Co.) and were then separated by 10% SDS-PAGE (Bio-Rad Laboratories, Inc.) and transferred onto PVDF membranes (MilliporeSigma) [27]. The membranes were then incubated with primary antibodies against GATA5 (dilution, 1:1,000), TMEM100 (dilution, 1:200; both from Santa Cruz Biotechnology, Inc.), MMP2 (dilution, 1:2,000; cat. no. ab92536), MMP9 (dilution, 1:1,000; cat. no. ab76003), E-cadherin (dilution, 1:1,000; cat. no. ab133597), N-cadherin (dilution, 1:1,000; cat. no. ab207608), Vimentin (dilution, 1:2,000; cat. no. ab16700), Snail (dilution, 1:1,000; cat. no. ab216347) and GAPDH (dilution, 1:2,500; cat. no. ab9485; all from Abcam) overnight at 4°C. The membranes were then washed with PBS-Tween 20 four times and incubated with a horseradish peroxidase-conjugated secondary antibody. Finally, the bands were visualized using an ECL detection system (Beyotime Institute of Biotechnology) and the density of the band was determined using ImageJ software (NIH, Bethesda, MD, USA)

### Statistical analysis

All statistical analyses were performed using SPSS 21.0 software (IBM Corp.). All data are expressed as the mean ± SD. The differences among multiple groups were analyzed using a one-way ANOVA followed by a Tukey’s post hoc test. The differences between two groups were compared using a two-tailed unpaired Student’s t-test. P < 0.05 was considered to indicate a statistically significant difference.

## Results

In this study, we explored the expression and functional roles of TMEM100 in PCa cells. The data showed that TMEM100 was low expressed in PCa and involved in PCa prognosis. In addition, TMEM100 overexpression repressed DU145 cell proliferation, igration and invasion, as well as EMT. Moreover, GATA5 was found to be downregulated in PCa and was positively associated with TMEM100 expression. GATA5 silencing had reversed effects on TMEM100-regulated proliferation, migration and EMT in DU145 cells.

### TMEM100 is downregulated and associated with poor prognosis in PCa

**Figure 1.**
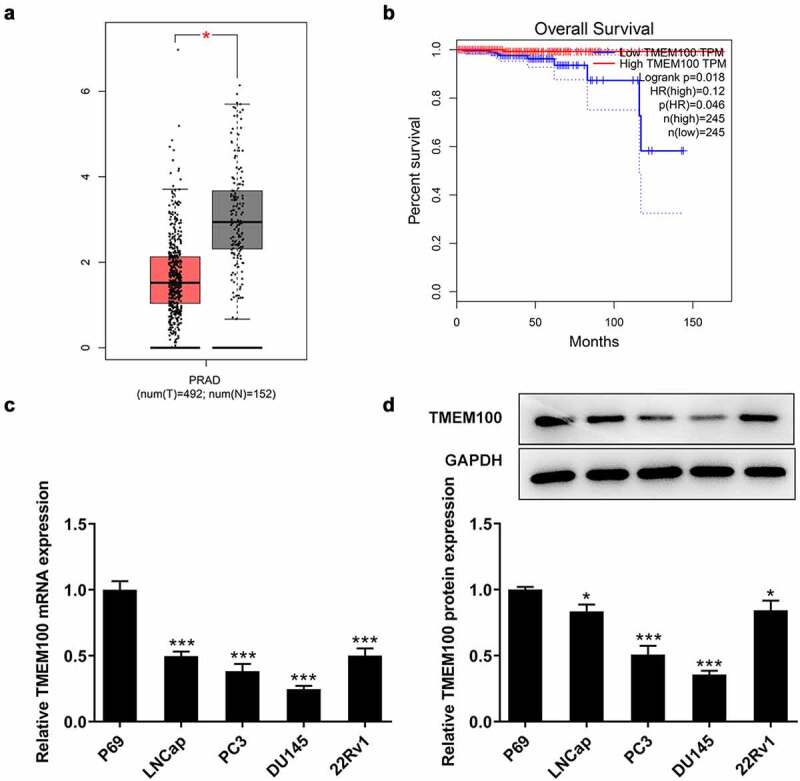
Downregulated TMEM100 expression is associated with poor prognosis in PCa. a, Data in GEPIA database showed downregulation of TMEM100 in PCa. b, Data in GEPIA database showed the relationship of TMEM100 expression and over survival of PCa patients. mRNA (c) and protein level (d) of TMEM100 in normal prostatic epithelial cells and several PCa cells were detected. Data are expressed as mean ± SD. *P < 0.05, ***P < 0.001 versus control.

Firstly, the differences in the expression levels of TMEM100 in PCa tissues and cells were investigated. As shown in Figure 1a, TMEM100 was markedly downregulated in PCa tissues according to the Gene Expression Profiling Interactive Analysis (GEPIA) database. In addition, the expression of TMEM100 in prostate tissue samples was associated with the overall survival (Figure 1b) in patients with PCa obtained from the GEPIA database. Furthermore, RT-qPCR and Western blot analysis revealed that the TMEM100 mRNA and protein levels were both significantly reduced in several PCa cell lines compared with those in the normal prostatic epithelial cell line, P69. Among these PCa cell lines, DU145 cells had the lowest TMEM100 expression. Therefore, DU145 cells were selected for use in the subsequent experiments (Figure 1c,d).

### TMEM100 overexpression attenuates the proliferation of DU145 cells

**Figure 2.**
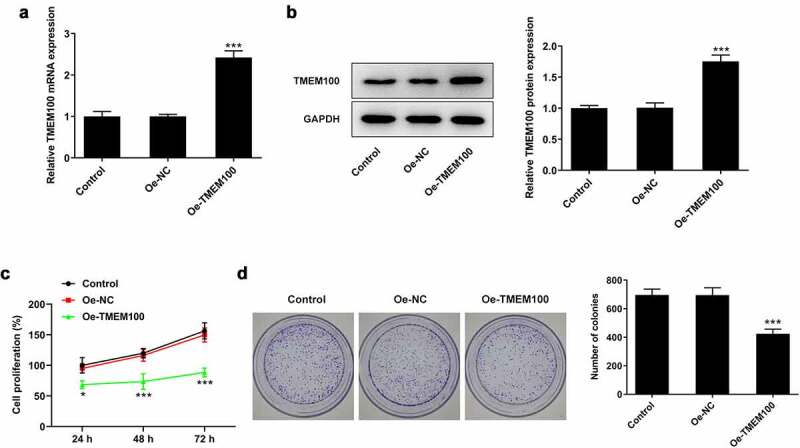
Effects of TMEM100 overexpression on the proliferation of DU145 cells. mRNA (a) and protein level (b) of TMEM100 were measured after TMEM100 was overexpressed. Cell proliferation was identified by CCK-8 assay (c) and colony formation assay (d). Data are expressed as mean ± SD. *P < 0.05, ***P < 0.001 versus NC group.

To explore the biological function of TMEM100 in PCa cells, DU145 cells were transfected with Oe-TMEM100 to overexpress TMEM100 and the transfection efficiency was then determined (Figure 2a,b). The effect of TMEM100 overexpression on DU145 cell proliferation was evaluated using CCK-8 and colony formation assays. As shown in Figure 2c, the proliferation of DU145 cells was significantly attenuated following TMEM100 overexpression compared with the negative (NC) group. Consistently, the number of cell colonies was notably decreased in the TMEM100 overexpression group compared with the NC group (Figure 2d).

### TMEM100 overexpression attenuates DU145 cell migration, invasion and EMT in PCa cells

**Figure 3.**
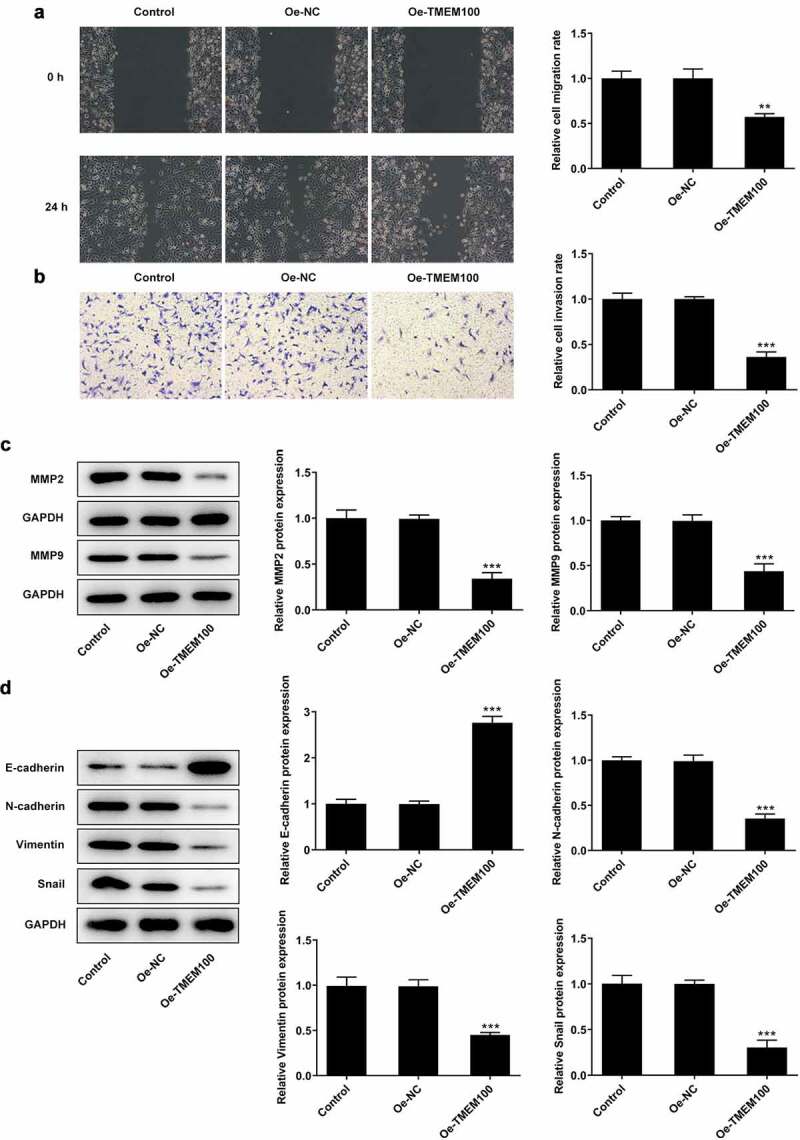
Effects of TMEM100 overexpression on migration, invasion and EMT of DU145 cells. A, Wound healing assay was performed to assess cell migration. B, Transwell assay was used to detect cell invasion. C, Protein levels of MMP2 and MMP9 were measured by Western blot assay. D, EMT-related proteins was identified by detection of E-cadherin, N-cadherin, Vimentin and Snail. Data are expressed as mean ± SD. *P < 0.05, **P < 0.01, ***P < 0.001 versus NC group.

To further investigate the effect of TMEM100 on cell invasion and migration, DU145 cells were transfected with Oe-TMEM100 to overexpress TMEM100. As shown in Figure 3a,b, the wound healing and Transwell assays revealed that TMEM100 overexpression attenuated the migration and invasion abilities of DU145 cells. Western blot analysis demonstrated that the expression levels of the invasion- and migration-related proteins, MMP2 and MMP9, were notably decreased compared with the control groups (Figure 3c). Furthermore, the protein expression levels of N-cadherin, Vimentin and Snail were increased, while those of E-cadherin were reduced following TMEM100 overexpression. The aforementioned findings suggested that TMEM100 exerted an inhibitory effect on the EMT process (Figure 3d).

Transcription factor GATA5 is associated with PCa

**Figure 4.**
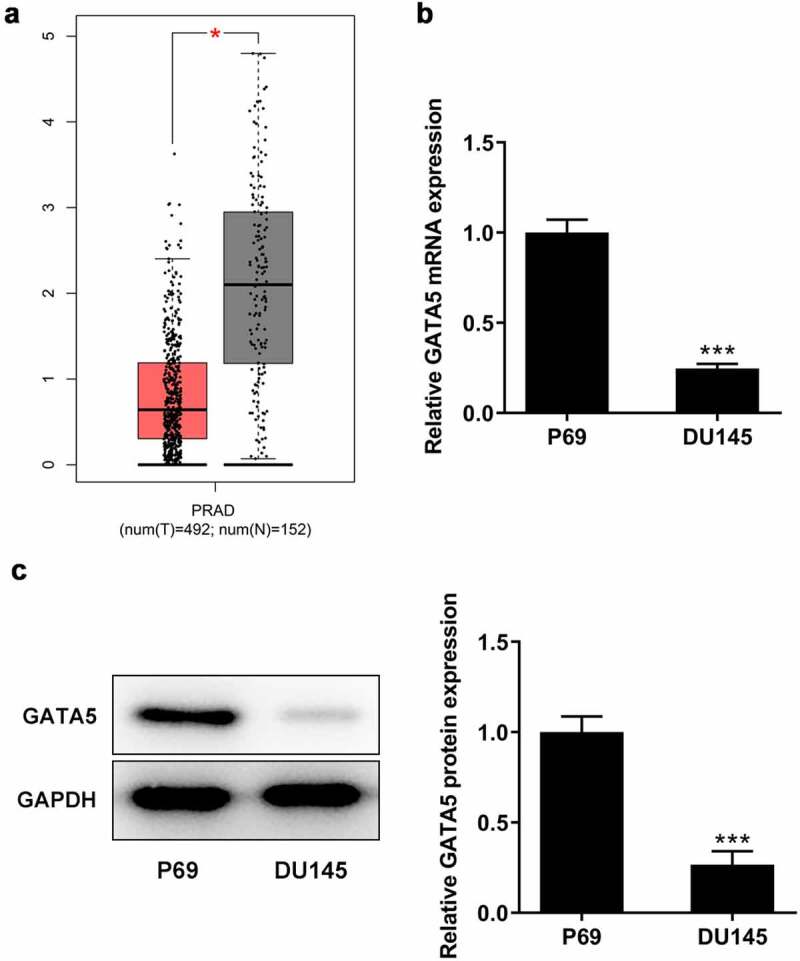
GATA5 expression is downregulated in PCa tissues and cells. A, Data in GEPIA database showed downregulation of GATA5 in PCa. mRNA (b) and protein levels (c) of GATA5 in normal prostatic epithelial cells and DU145 cells were detected. Data are expressed as mean ± SD. *P < 0.05, ***P < 0.001 versus control.

As shown in Figure 4a, using the data from the GEPIA database, GATA5 was found to be significantly downregulated in PCa tissues compared with normal prostate tissues. Consistent with the aforementioned findings, the mRNA and protein expression levels of GATA5 were considerably decreased in DU145 cells compared with the normal control P69 cells (Figure 4b,c). These results indicated that GATA5 may be involved in PCa progression.

### GATA5 binds to the TMEM100 promoter to modulate TMEM100 expression

**Figure 5.**
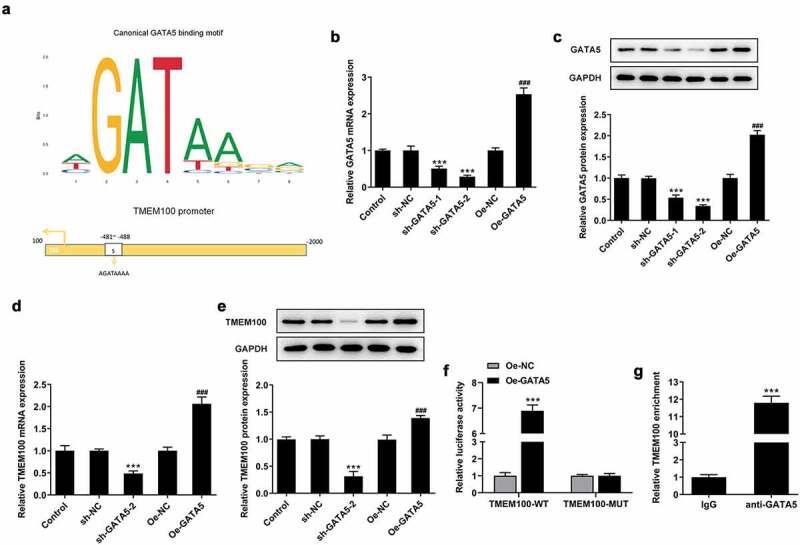
GATA5 binds to the TMEM100 promoter and regulates TMEM100 expression. a, The binding site of GATA5 and TMEM100. mRNA (b) and protein level (c) of GATA5 were measured after GATA5 was overexpressed or silenced. mRNA (d) and protein level (e) of TMEM100 were detected after GATA5 was overexpressed or silenced. ***P < 0.001 versus sh-NC. F, TMEM100 promoter activity was evaluated by luciferase reporter assay. G, Chromatin immunoprecipitation assay was carried out tom detect the binding level of GATA5 and TMEM100 promotor. Data are expressed as mean ± SD. ***P < 0.001 versus control. ^###^P < 0.001 versus Oe-NC.

Bioinformatics analysis using the JASPAR database predicted that GATA5 could bind to the TMEM100 promoter (Figure 5a). To verify the interaction between GATA5 and TMEM100, DU145 cells were transfected with Oe-GATA5 or sh-GATA5 to overexpress or knockdown GATA5, respectively. The cell transfection efficiency was determined using RT-qPCR and Western blot analysis (Figure 5b,c). Between the two shRNAs targeting GATA5, sh-GATA5-2 displayed a more potent silencing effect on GATA5 expression and it was therefore selected for the subsequent experiments. The results demonstrated that the expression of TMEM100 was significantly decreased in cells transfected with sh-GATA5-2 compared with control cells. By contrast, GATA5 overexpression enhanced TMEM100 expression in DU145 cells (Figure 5d,e). Furthermore, the results of the dual luciferase reporter assay showed that the luciferase activity in cells transfected with the TMEM100-WT reporter plasmid was markedly enhanced following co-transfection with Oe-GATA5. However, no obvious changes in the luciferase activity were observed in the other groups, indicating that the activity of the TMEM100 promoter was enhanced by GATA5 overexpression (figure 5f). Similarly, ChIP assays revealed that TMEM100 was significantly enriched in the GATA5 group compared with the IgG group (Figure 5g). The aforementioned results verified the binding capacity of GATA5 on the TMEM100 promoter and the regulatory effect of GATA5 on the expression of TMEM100.

### Interference of GATA5 reverses the effects of TMEM100 overexpression on the proliferation, migration and EMT in PCa cells

**Figure 6.**
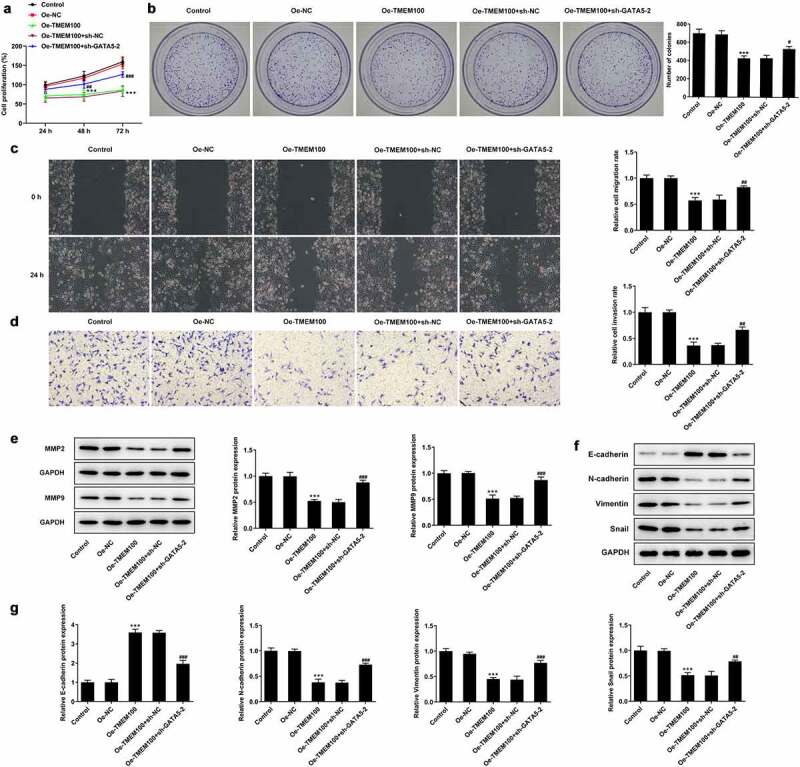
Effects of GATA5 silencing on TMEM100-mediated migration, invasion and EMT of DU145 cells. Cell proliferation was identified by CCK-8 assay (a) and colony formation assay (b). C, Wound healing assay was implemented to assess cell migration. D, Transwell assay was employed to detect cell invasion. E, Protein levels of MMP2 and MMP9 were investigated by Western blot assay. F and G, EMT-related proteins was determined with detection of E-cadherin, N-cadherin, Vimentin and Snail. Data are expressed as mean ± SD. ***P < 0.001 versus Oe-NC. ^#^P < 0.05, ^##^P < 0.01, ^###^P < 0.001 versus Oe-TMEM100+ sh-NC.

Finally, the current study aimed to elucidate whether TMEM100 could regulate the proliferation, migration and EMT in PCa cells via interacting with GATA5. CCK-8 and colony formation assays showed that the cell proliferation rate was evidently attenuated in cells overexpressing TMEM100, while GATA5 silencing reversed the inhibitory effect of TMEM100 on DU145 cell proliferation (Figure 6a,b). As shown in Figure 6c,d, the migration and invasion capacities of DU145 cells were attenuated by TMEM100 overexpression compared with the Oe-NC group. However, GATA5 knockdown enhanced the migration and invasion abilities of TMEM100-overexpressing DU145 cells. Consistently, the protein expression levels of MMP2 and MMP9 were increased in cells transfected with sh-GATA5 compared with those overexpressing TMEM100 (Figure 6e). Additionally, Western blot results revealed that the protein expression levels of N-cadherin, Vimentin and Snail were reduced, while those of E-cadherin were elevated by TMEM100 overexpression. However, GATA5 silencing abrogated the effect of TMEM100 on the protein expression levels of N-cadherin, Vimentin, Snail and E-cadherin, supporting the involvement of GATA5 in the TMEM100-mediated EMT process. These findings implied that GATA5 silencing could reverse the inhibitory effects of TMEM100 overexpression on the proliferation, migration and EMT in PCa cells.

## Discussion

PCa is one of the most common male malignant diseases. However, its treatment options remain limited due to the lack of effective therapy approaches for treating PCa at an advanced stage of the disease [28–30]. At present, gene therapy has been considered as an appealing treatment option for PCa [31]. The present study demonstrated that TMEM100 expression was decreased in PCa tissues and cells and downregulated TMEM100 was associated with poor prognosis. Furthermore, TMEM100 overexpression attenuated the cell proliferation, migration and EMT in PCa cells. In addition, the results showed that GATA5 could transactivate TMEM100 expression, while GATA5 knockdown reversed the effects of TMEM100 on the behavior of PCa cells.

TMEM100 was identified as a potentiating modulator of transient receptor potential ankyrin 1-V1 complexes, while in 2001, it was identified as a novel transcript from the mouse genome [32]. Previous studies revealed that TMEM100 was involved in apoptosis, angiogenesis and cancer development [33–35]. Additionally, a study demonstrated that the expression of TMEM100 was elevated in the transition zone of the prostate compared with that in the peripheral zone in aggressive tumors [36]. Herein, bioinformatics analysis using the GEPIA database showed that TMEM100 was downregulated in PCa tissues, and its reduced expression was associated with decreased overall survival in patients with PCa. Consistently, the expression of TMEM100 was decreased in several PCa cell lines compared with normal prostatic epithelial cells. Furthermore, TMEM10 overexpression attenuated PCa cell proliferation, migration and invasion. This finding was consistent with the results of previous studies, suggesting that TMEM100 exhibited tumor-suppressive activity in several types of cancers [12,37,38].

GATA5 is one of the members of the GATA-binding protein family (GATA1-6) consisting of transcription factors that bind to the DNA consensus sequence GATA [13]. GATA5 is involved in the morphogenesis of organs derived from the mesoderm or endoderm, including the lungs, gut, heart and liver [39]. Furthermore, GATA5 is associated with malignant transformation in several types of human cancers [14,40,41]. For instance, GATA5 overexpression restrained the growth and metastasis of cholangiocarcinoma cells, while GATA5 silencing enhanced cell proliferation and colony formation in hepatocellular carcinoma cells [16,17].

To investigate the interaction of TMEM100 with GATA5, the binding capacity of GATA5 on TMEM100 promoter was predicted using the JASPAR database. Subsequently, the interaction between TMEM100 and GATA5 was confirmed using dual luciferase reporter and ChIP assays. Furthermore, functional experiments demonstrated that GATA5 silencing in cells overexpressing TMEM100 promoted TMEM100-mediated DU145 cell proliferation, migration, invasion and EMT. These findings indicated that GATA5 could inhibit PCa tumorigenesis and metastasis via transactivating TMEM100.

However, there are several limitation in this study. Firstly, we found that the levels of TMEM100 were higher in androgen receptor (AR)-positive PCa cell lines (LNCaP and 22RV1) than that of AR-negative cell lines (PC-3 and DU145, Figure 1), thus we speculate that AR signaling may play a role in TMEM100/GATA5 interaction in PCa. Secondly, DU145 cell line was the only one to be used for overexpressing TMEM100 due to its low level of TMEM100, we are exploring biological effects of TMEM100 in other PCa cell lines and investigating other potential mechanism in our next study. Thirdly, MMP2/9, Snail and other EMT markers were found to affect the GATA5-TMEM100 pathway, so the regulatory mechanism of TMEM100 needs to be explained. Finally, we did not explore the relationship between TMEM100 and PCa Grade in this study, and we will collect clinical tissues from patients with different stages and grades of PCa to investigate the role of TMEM10 in different stages and grades of PCa in further study.

## Conclusion

In conclusion, the present study provided novel insights into the role of GATA5 on the oncogenic activity and metastasis of PCa cells. GATA5 could attenuate PCa cell proliferation, invasion and metastasis rates via transactivating TMEM100, highlighting the potential therapeutic value of GATA5 and TMEM100 in treating PCa.

## Highlights

1. TMEM100 is downregulated in prostatic cancer and is associated with poor prognosis of patients with prostatic cancer.
2. Overexpression of TMEM100 inhibits prostatic cancer cell proliferation, migration, invasion and EMT.
3. TMEM100 is regulated by GATA5 transcriptionally.
4. GATA5 silencing reversed effects of TMEM100 on prostatic cancer cell behaviors.
